# Impact of nutritional stress on drug susceptibility and biofilm structures of *Burkholderia pseudomallei* and *Burkholderia thailandensis* grown in static and microfluidic systems

**DOI:** 10.1371/journal.pone.0194946

**Published:** 2018-03-26

**Authors:** Chitchanok Anutrakunchai, Jan G. M. Bolscher, Bastiaan P. Krom, Sakawrat Kanthawong, Sorujsiri Chareonsudjai, Suwimol Taweechaisupapong

**Affiliations:** 1 Biofilm Research Group, Faculty of Dentistry, Khon Kaen University, Khon Kaen, Thailand; 2 Melioidosis Research Center, Khon Kaen University, Khon Kaen, Thailand; 3 Department of Oral Biochemistry, Academic Centre for Dentistry Amsterdam, University of Amsterdam and Vrije Universiteit Amsterdam, Amsterdam, The Netherlands; 4 Department of Preventive Dentistry, Academic Centre for Dentistry Amsterdam, University of Amsterdam and Vrije Universiteit Amsterdam, Amsterdam, The Netherlands; 5 Department of Microbiology, Faculty of Medicine, Khon Kaen University, Khon Kaen, Thailand; J Craig Venter Institute, UNITED STATES

## Abstract

*Burkholderia pseudomallei* is the causative agent of melioidosis and regarded as a bioterrorism threat. It can adapt to the nutrient-limited environment as the bacteria can survive in triple distilled water for 16 years. Moreover, *B*. *pseudomallei* exhibits intrinsic resistance to diverse groups of antibiotics in particular while growing in biofilms. Recently, nutrient-limited condition influenced both biofilm formation and ceftazidime (CAZ) tolerance of *B*. *pseudomallei* were found. However, there is no information about how nutrient-limitation together with antibiotics used in melioidosis treatment affects the structure of the biofilm produced by *B*. *pseudomallei*. Moreover, no comparative study to investigate the biofilm architectures of *B*. *pseudomallei* and the related *B*. *thailandensis* under different nutrient concentrations has been reported. Therefore, this study aims to provide new information on the effects of four antibiotics used in melioidosis treatment, *viz*. ceftazidime (CAZ), imipenem (IMI), meropenem (MEM) and doxycycline (DOX) on biofilm architecture of *B*. *pseudomallei* and *B*. *thailandensis* with different nutrient concentrations under static and flow conditions using confocal laser scanning microscopy. Impact of nutritional stress on drug susceptibility of *B*. *pseudomallei* and *B*. *thailandensis* grown planktonically or as biofilm was also evaluated. The findings of this study indicate that nutrient-limited environment enhanced survival of *B*. *pseudomallei* in biofilm after exposure to the tested antibiotics. The shedding planktonic *B*. *pseudomallei* and *B*. *thailandensis* were also found to have increased CAZ tolerance in nutrient-limited environment. However, killing activities of MEM and IMI were stronger than CAZ and DOX on *B*. *pseudomallei* and *B*. *thailandensis* both in planktonic cells and in 2-day old biofilm. In addition, MEM and IMI were able to inhibit *B*. *pseudomallei* and *B*. *thailandensis* biofilm formation to a larger extend compared to CAZ and DOX. Differences in biofilm architecture were observed for biofilms grown under static and flow conditions. Under static conditions, biofilms grown in full strength modified Vogel and Bonner’s medium (MVBM) showed honeycomb-like architecture while a knitted-like structure was observed under limited nutrient condition (0.1×MVBM). Under flow conditions, biofilms grown in MVBM showed a multilayer structure while merely dispersed bacteria were found when grown in 0.1×MVBM. Altogether, this study provides more insight on the effect of four antibiotics against *B*. *pseudomallei* and *B*. *thailandensis* in biofilm under different nutrient and flow conditions. Since biofilm formation is believed to be involved in disease relapse, MEM and IMI may be better therapeutic options than CAZ for melioidosis treatment.

## Introduction

*Burkholderia pseudomallei* is the etiological agent of melioidosis, an emerging infectious disease associated with high morbidity and mortality [1,2]. *B*. *pseudomallei* exhibits intrinsic resistance to diverse groups of antibiotics and is even more resistant when growing in biofilms, leading to treatment difficulties [3–5]. Furthermore, the disease has a high relapse rate despite appropriate antibiotic therapy [6]. Relapsing melioidosis correlates with biofilm formation [7]. Treatment recommendations are derived from the outcomes of a series of clinical trials conducted in endemic regions, mostly Thailand and northern Australia, and is summarized in several studies [8–10]. Ceftazidime (CAZ) and carbapenems, such as imipenem (IMI) and meropenem (MEM), remain the backbone of current initial acute-phase therapy for melioidosis [8]. Doxycycline (DOX) was previously recommended for postexposure prophylaxis [11] and is the second choice for eradication therapy [12]. Different antibiotic regimens have been used by different groups to treat melioidosis, and many unanswered questions remain.

*B*. *pseudomallei* has been reported to form biofilm both *in vitro* and *in vivo* [3,13–15]. We previously demonstrated several factors affecting *B*. *pseudomallei* biofilm production including the nutrient concentration in the medium [16–18]. The effect of nutrient concentration on biofilm production has been reported for several species of bacteria [16,17,19–22]. The results are controversial, with some studies indicating that biofilm production was enhanced in nutrient-limited condition [16,17,19–21] and others finding that biofilm formation was reduced (such as for *Listeria monocytogenes*) [22,23]. Recently, we found that nutrient-limited condition induces biofilm formation concomitantly with CAZ tolerance in *B*. *pseudomallei* [16,17]. However, there is no information about how this relates to each other. In this study, the effects of nutrient limitation on the susceptibility of *B*. *pseudomallei* and *B*. *thailandensis* to CAZ, IMI, MEM and DOX was studied in planktonic and biofilm cultures. Biofilm architecture of *B*. *pseudomallei* and *B*. *thailandensis* grown under static and flow conditions was determined using confocal laser scanning microscopy (CLSM). *B*. *thailandensis*, a non-virulent biotype of *B*. *pseudomallei*, was also used in the present study because it has been used previously as a model to study certain aspects of *B*. *pseudomallei* biology and does not require a biosafety level 3 containment facility. Therefore, *B*. *thailandensis* was used for evaluation of the effect of CAZ on biofilm formation under flow conditions using a Bioflux microfluidics system. The results demonstrated that IMI and MEM offered not only stronger eradicating effect on *B*. *pseudomallei* and *B*. *thailandensis* grown planktonically and in 2-day old biofilm but also reduced levels of *B*. *pseudomallei* biofilm formation in comparison to CAZ and DOX.

## Materials and methods

### Bacterial strains and growth conditions

The *B*. *pseudomallei* K96243 and *B*. *thailandensis* E264 were used. A preculture was obtained by inoculating 10 ml of nutrient broth (Criterion, Hardy Diagnostics, CA) with a single colony of bacteria initially grown on nutrient agar (NA) (Criterion). The bacterial cultures were incubated overnight at 37°C in a 200 rpm shaker-incubator and used as inoculum in all experiments. Biofilm formation was studied in full strength modified Vogel and Bonner’s medium (MVBM) [24] and in 10-fold diluted of MVBM (0.1×MVBM).

## Growth rate measurement

The growth curve of *B*. *pseudomallei* K96243 and *B*. *thailandensis* E264 in either MVBM or 0.1×MVBM was determined using a computerized spectrophotometric incubator (Varioskan Flash, Thermo Fisher Scientific, MA) as previous report with some slight modification [16]. The bacteria in nutrient broth from an overnight culture were washed and re-suspended with phosphate buffered saline (PBS) to an optical density measured at 600 nm (OD_600_) of 0.2 and diluted 1:50 in fresh medium or PBS. Then, 200 μl of each bacterial inoculum was added into 6 wells of a sterile 96-well round-bottomed plastic tissue culture plate. Wells containing only medium served as a negative control. The microtiter plate was placed in a computerized spectrophotometric incubator and incubated aerobically at 37°C. Growth of bacterial cells was monitored in terms of the change in the turbidity (OD_600_), at 60-min intervals, for a period of 72 h. All experiments were repeated on two separate occasions in sextuplicate. Growth rate of *B*. *pseudomallei* K96243 and *B*. *thailandensis* E264 in either MVBM or 0.1×MVBM was determined from the exponential growth phase as Eq (1); OD_1_ and OD_2_ are optical density at time t_2_ and t_1_, respectively [25,26]. Then the doubling or generation times was calculated from growth rate as Eq (2) [27].

$$growth\;rate\;\left(h^{-1}\right)=\frac{\left(lnOD_{2}-lnOD_{1}\right)}{\left(t_{2}-t_{1}\right)}$$ (1)

$$doubling\;time\;\left(h\right)=\frac{ln2}{generation\;time}$$ (2)

### Drug susceptibility testing of planktonic bacteria

The antibiotics used in this study were CAZ, IMI, MEM and DOX. The minimum inhibitory concentration (MIC) and the minimum bactericidal concentration (MBC) of the drugs were determined by the broth microdilution method [28]. Briefly, cultures of each bacterial strain were adjusted to approximately 1×10^6^ CFU/ml in Müller Hilton broth (MHB) (Criterion). The antibiotics were 2-fold serially diluted in MHB with a final volume of 50 μl in each well of a 96-well microtiter plate. The final concentration of antibiotics ranged from 0.0625 to 512 μg/ml. Then 50 μl of the bacterial suspensions was added to the test wells which gave a final bacterial density of approximately 5×10^4^ CFU/well. Wells containing only media and culture-free antibiotics were included as negative controls. The plates were incubated at 37°C for 24 h. Subsequently, bacterial growth was examined and the lowest concentration of antibiotics negative for visible growth was recorded as the MIC. Subsequently, 10 μl aliquots, taken from wells showing no visible growth, were placed onto the surface of NA. The lowest concentration of antibiotics negative for subsequent growth was recorded as the MBC. All experiments were repeated on three separate occasions in triplicate.

### Drug susceptibility testing of bacterial biofilm

Drug susceptibility testing of *B*. *pseudomallei* K96243 and *B*. *thailandensis* E264 biofilms in MVBM and 0.1×MVBM media was determined using the Calgary biofilm device (CBD) as previously described [16] with some slight modification. The CBD is the rapid and reproducible assay of biofilm susceptibilities to antibiotics [29]. It consists of a lid with 96 pegs (Nunc, Thermo Fisher Scientific, Denmark) that can be placed on top of a standard 96-well microtiter plate. From the study of Ceri et al using CBD, no significant difference (*P* > 0.1) was seen between biofilms formed on each of the 96 pegs [29]. Therefore, 96 equivalent biofilms could be tested with 4 antibiotics to demonstrate the inhibitory or killing activity of each antibiotic.

To allow bacterial adhesion to each peg, a final volume (100 μl) of a standardized bacterial suspension in PBS was added in each well of 96-well plate and incubated at 37°C for 2 h. Wells containing PBS alone served as the negative controls. To grow initial biofilms, the pegs were placed onto a new 96-well plate containing fresh medium, either MVBM or 0.1×MVBM, and incubated at 37°C for 6 h. Then biofilms formed on the pegs of the CBD were transferred to a new 96-well plate which contained 100 μl/well of the 2-fold serial dilution of antibiotics in either MVBM or 0.1×MVBM. The final concentrations of antibiotics were ranging from 0.0625 to 512 μg/ml. Antibiotic-free wells were also included as growth control. The plates were incubated at 37°C for 24 h. The lids were removed and the wells were checked for turbidity using a microplate reader at 620 nm to determine MIC values of antibiotics against shedding planktonic bacteria. Subsequently aliquots of the mixture of the antibiotics and the bacteria in each well were inoculated onto the surface of NA. The lowest concentration of the antibiotics negative for growth of the bacteria were recorded as the shedding planktonic MBC.

To determine minimum biofilm eliminating concentration (MBEC), the pegs containing biofilms were rinsed with PBS for 1 min and placed in a second 96-well plate containing MHB. The biofilms were removed from the pegs by sonication for 5 min. A new plate cover was added, and the viability of the biofilms was determined after 24 h of incubation at 37°C by reading the turbidity at 620 nm in a microplate reader. The MBEC is defined as the minimum concentration of antibiotic that inhibits regrowth (OD_620_ < 0.1) of biofilm bacteria in the recovery media. All experiments were repeated on three separate occasions in triplicate.

### Effects of drugs on biofilm formation, biofilm structures and viability of bacteria in static condition

To determine the effects of 4 antibiotics on biofilm formation and biofilm structures of *B*. *pseudomallei* K96243 or *B*. *thailandensis* E264, a subcidal concentration (16×MIC) of each antibiotic against biofilm bacteria was chosen. The Amsterdam Active Attachment (AAA) model [30,31] was used to culture biofilm under static condition. This biofilm model consists of stainless steel lid with clamps that contain 12 mm diameter round glass coverslips which were used as substrata to grow biofilms. The lid fits onto standard polystyrene 24-well plates (Greiner Bio-One, Austria). After assembling the lid and glass coverslip, the model was autoclaved. To allow adhesion on each glass coverslips, 1 ml of a standardized bacterial suspension (OD_600_ of 0.2) in PBS was added in each well of 24-well plate and incubated at 37°C for 2 h. After 2 h, the lid was transferred to a new plate containing fresh media (MVBM or 0.1×MVBM) twice daily and incubated aerobically at 37°C to allow biofilm formation until 32 h. Subsequently, biofilms were exposed to 16×MIC of each antibiotic (64 μg/ml CAZ, 16 μg/ml IMI, 32 μg/ml MEM and 32 μg/ml DOX) at 37°C for 16 h. Biofilms grown in media without antibiotics served as no-treatment controls. The glass coverslips in wells containing only the medium served as negative controls.

Biofilm formation was estimated using a crystal violet staining method as previously described [32] with some slight modification. After 48 h, glass coverslips with biofilms were transferred to a new plate containing 0.01% crystal violet solution. After 5 min, the biofilms were washed twice with water to remove the excess stains. Crystal violet bound to the biofilm on the glass coverslips was extracted with 1 ml/well of 2% sodium deoxycholate by shaking at 100 rpm for 5 min. Subsequently, the absorbance was measured at 620 nm using a microplate reader (Sunrise^™^, Tecan Trading AG, Switzerland). All experiments were performed on three separate occasions in quadruplicate.

To determine the effect of antibiotics on biofilm structure of *B*. *pseudomallei* K96243 and *B*. *thailandensis* E264 in the AAA-model, CLSM was used. After exposure to 16×MIC of each antibiotic, the 2-day old biofilm on the glass coverslips was fixed with 1% glutaraldehyde for 30 min at 4°C. The extracellular polymeric substance of the biofilm was stained with fluorescein isothiocyanate-concanavalin A (Sigma, Sigma-Aldrich, MO) for 5 min and washed with water to remove excess dye. The bacterial cells in biofilm were stained with 5 μg/ml of propidium iodide (Invitrogen, Thermo Fisher Scientific, CA) for 5 min and washed 3 times with water. The coverslips were examined using CLSM at 100× and 630× magnification (Zeiss LSM 800, Zeiss, Germany) and the biofilm structures were analyzed using the ZEN image software (Zeiss).

To determine the viability of *B*. *pseudomallei* K96243 and *B*. *thailandensis* E264 after exposed to 16×MIC of each antibiotic for 16 h, the 2-day old biofilm on the glass coverslips were submerged in 1.5 ml staining solution of LIVE/DEAD BacLight Bacterial Viability kit (Invitrogen) and incubated at 4°C in the dark for 15 min. Thereafter, it was fixed with 1% glutaraldehyde at 4°C for 30 min. The 2-day old biofilm was examined using CLSM and quantification of bacterial viability was determined using the ZEN image software.

### Effect of CAZ on biofilm formation of *B*. *thailandensis* in Bioflux microfluidics system

*B*. *thailandensis* E264 was chosen for further evaluation of the effect of CAZ on biofilm formation under flow conditions using a Bioflux microfluidics system (Bioflux Z1000, Fluxion Biosciences, CA) because it does not require a biosafety level 3 containment facility. The Bioflux Z1000 system with 48-well plate was used for biofilm formation basically as described previously [33]. Briefly, the microfluidic channels were inoculated with the bacterial suspension (OD_600_ = 0.2) in PBS from the outlet well to retain sterility upstream of the channel. Bacteria were allowed to adhere without flow at 37°C for 2 h. To initiate growth of biofilms, fresh medium was flowed from inlet well through the channel to outlet well at a flow rate of 0.5 dyn/cm^2^ at 37°C for 6 h similar as in static biofilm. Then the 16×MIC of CAZ mixed with medium was flowed through the channel for an additional 16 h at a flow rate of 0.5 dyn/cm^2^ (24 h-biofilm). Biofilms grown in media without the CAZ served as control. To obtain time course information on biofilm formation, images were captured at 3 different locations per channel every 10 min for a total of 24 h with 200× magnification using automated bright-field microscopy (Axio Observer Z1, Zeiss, Germany). The images were analyzed using Fiji platform [34].

### Statistical analysis

Comparisons of biofilm formation and live/dead ratio among the tested groups in each medium were conducted with the Kruskal-Wallis and Dunn’s multiple comparison tests using GraphPad Prism 5 (GraphPad Software, Inc., CA, USA). The level required for statistical significance was *p* < 0.01.

## Results

### Nutrient-limited environment retards the growth but enhances CAZ tolerance of shedding planktonic *B*. *pseudomallei* and *B*. *thailandensis*

**Fig 1:**
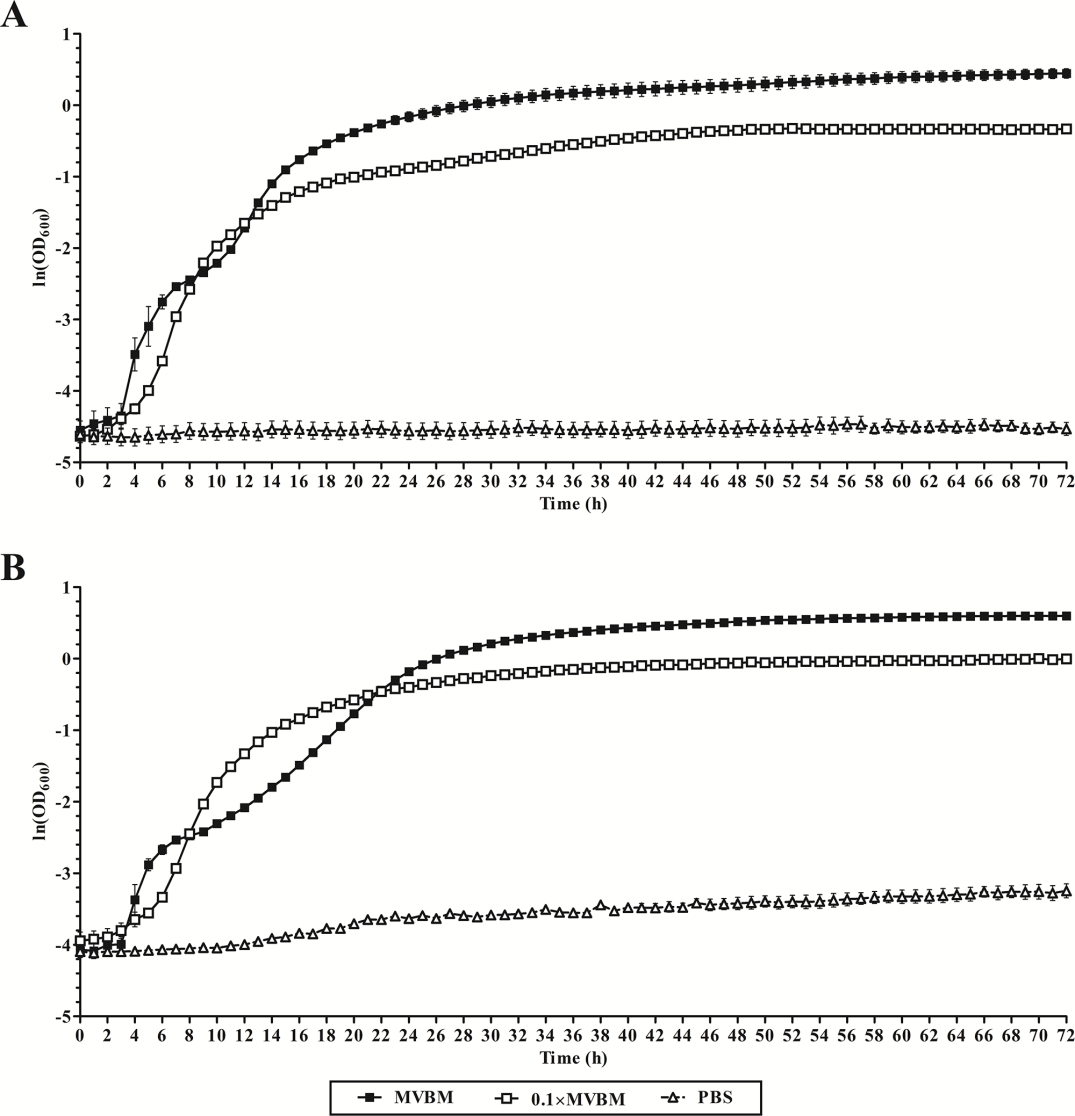
Planktonic bacterial growth curve. Growth curve of *B*. *pseudomallei* K96243 (A) and *B*. *thailandensis* E264 (B) cultured in MVBM, 0.1×MVBM and PBS at 37°C for 72 h. Data are the mean value of two independent experiments carried out in sextuplicate.

The growth curves of *B*. *pseudomallei* K96243 (Fig 1A) and *B*. *thailandensis* E264 (Fig 1B) showed that growth rates of both strains in the nutrient-limited condition (0.1×MVBM) were lower than in MVBM. The estimated doubling times of *B*. *pseudomallei* K96243 in MVBM and 0.1×MVBM were 67 and 73 mins, respectively. The doubling times of *B*. *thailandensis* E264 in MVBM and 0.1×MVBM were 75 and 94 mins, respectively. Both strains did not show growth in PBS. The results demonstrated that nutrient shortage distress the bacterial growth.

The MIC and MBC determinations of planktonic *B*. *thailandensis* E264 and *B*. *pseudomallei* K96243 revealed that IMI and MEM exhibited higher killing activities than CAZ and DOX to both bacterial strains whereas DOX exhibited the lowest killing activity (Table 1). When these bacteria were grown as biofilms using CBD, the shedding planktonic MBC of all tested antibiotics in each media (Table 2) were higher than the MBC values shown in Table 1. Notably, the shedding planktonic MIC values of CAZ against both strains in 0.1×MVBM were much higher than those in MVBM, contrary to all other tested antibiotics. Moreover, the antibiotic susceptibilities of 6-h old biofilm bacteria (MBEC results) in both media were much higher than those of planktonic cells (both MBC and shedding planktonic MBC results).

**Table 1:** Minimum inhibitory concentration (MIC) and minimum bactericidal concentration (MBC) of the tested antibiotics against planktonic *B*. *pseudomallei* K96243 and *B*. *thailandensis* E264.

Antibiotic	*B*. *pseudomallei* K96243		*B*. *thailandensis* E264	
	MIC (μg/ml)	MBC (μg/ml)	MIC (μg/ml)	MBC (μg/ml)
**CAZ**	4	16	4	8
**IMI**	1	2	1	2
**MEM**	2	4	2	4
**DOX**	2	32	2	64

CAZ: ceftazidime, IMI: imipenem, MEM: meropenem, DOX: doxycycline

**Table 2:** Susceptibility of *B*. *pseudomallei* K96243 and *B*. *thailandensis* E264 in planktonic and biofilm forms to the tested antibiotics determined by Calgary biofilm device.

Medium	Antibiotic	*B*. *pseudomallei* K96243			*B*. *thailandensis* E264		
		PMIC (μg/ml)	PMBC (μg/ml)	MBEC (μg/ml)	PMIC (μg/ml)	PMBC (μg/ml)	MBEC (μg/ml)
**MVBM**	CAZ	8	128	> 512	8	64	> 512
	IMI	0.5	64	> 512	0.25	16	> 512
	MEM	1	128	> 512	1	64	> 512
	DOX	16	> 512	> 512	32	> 512	> 512
**0.1×MVBM**	CAZ	32	128	> 512	64	64	> 512
	IMI	0.25	32	> 512	0.25	16	> 512
	MEM	0.25	128	> 512	0.25	32	> 512
	DOX	8	> 512	> 512	16	> 512	> 512

CAZ: ceftazidime, IMI: imipenem, MEM: meropenem, DOX: doxycycline

MVBM: modified Vogel and Bonner’s medium, 0.1×MVBM: 10-fold dilution of modified Vogel and Bonner’s medium

PMIC: shedding planktonic minimum inhibitory concentration, PMBC: shedding planktonic minimum bactericidal concentration, MBEC: minimum biofilm eliminating concentration

### Nutrient concentration and antibiotics transformed biofilm-forming capacity

**Fig 2:**
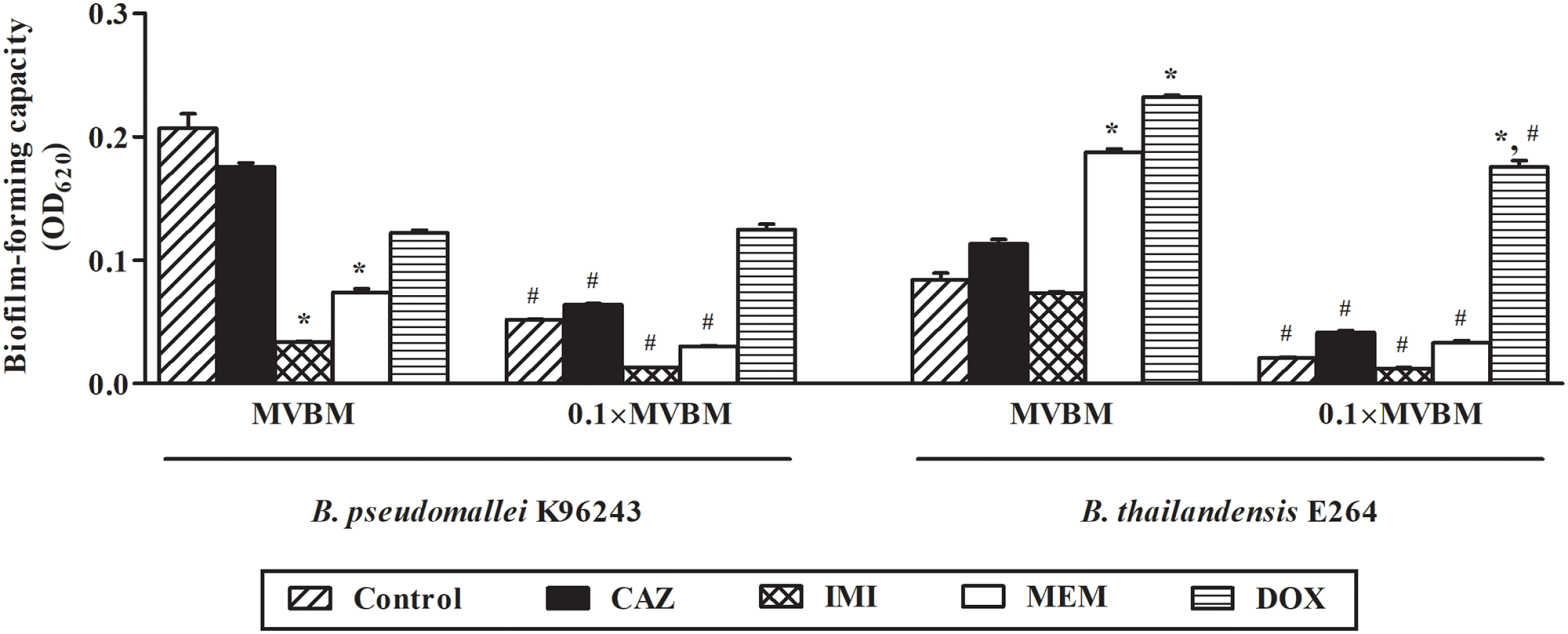
Biofilm-forming capacity of *B*. *pseudomallei* K96243 and *B*. *thailandensis* E264 after exposed to 16×MIC of each antibiotic in MVBM and 0.1×MVBM for 16 h. OD_620_ of the biofilm after staining with crystal violet. Data are the mean value of two independent experiments carried out in quadruplicate. *p < 0.01 compared to control in the same medium. ^#^*p* < 0.01 compared to the same antibiotic in MVBM.

The effect of nutrient concentration and antibiotics on biofilm-forming capacity of the tested bacteria are shown in Fig 2. In MVBM, the biofilm-forming capacity of *B*. *pseudomallei* K96243 was significantly reduced after exposed to either IMI or MEM compared with control (*p* < 0.01) while there was no significant reduction of biofilm upon exposure to CAZ or DOX. On the contrary, higher biofilm formation of *B*. *thailandensis* E264 was observed after exposed to CAZ, MEM or DOX in both MVBM or 0.1×MVBM compared with the untreated control. However, the biofilm-forming capacity of both strains in 10-fold diluted medium (0.1×MVBM) of all conditions (with and without antibiotics) was lower than those in MVBM. Among all tested antibiotics, IMI was the most effective antibiotic in inhibiting biofilm formation of *B*. *pseudomallei* and *B*. *thailandensis* in either MVBM or 0.1×MVBM.

### Phenotypic divergence of *B*. *pseudomallei* and *B*. *thailandensis* biofilm structure after treated with antibiotics

**Fig 3:**
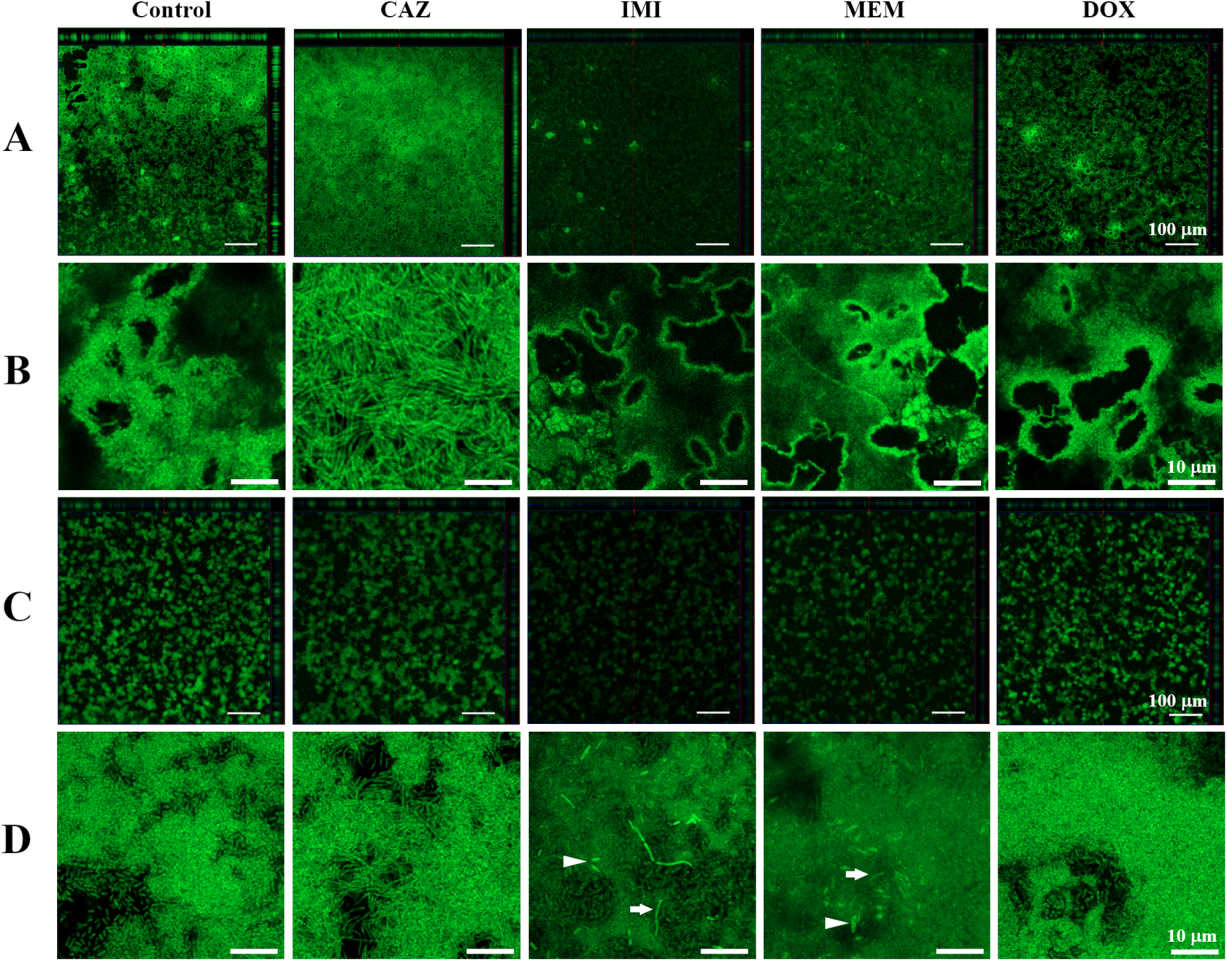
Effect of antibiotics on biofilm structure of *B*. *pseudomallei*. CLSM images of 2-day old biofilm of *B*. *pseudomallei* K96243 exposed to 16×MIC of each antibiotic in MVBM (A, B) and 0.1×MVBM (C, D) for 16 h and stained with fluorescein isothiocyanate-concanavalin A. A representative CLSM image is shown for each sample. At higher magnification (B, D), CAZ-treated biofilm cells displayed a long filamentous change while short filaments (white arrow) and large ovoid cells (white arrow head) were observed for IMI- and MEM-treated biofilm cells in 0.1×MVBM. The scale bar at 100× magnification (A, C): 100 μm; the scale bar at 630× magnification (B, D): 10 μm.

**Fig 4:**
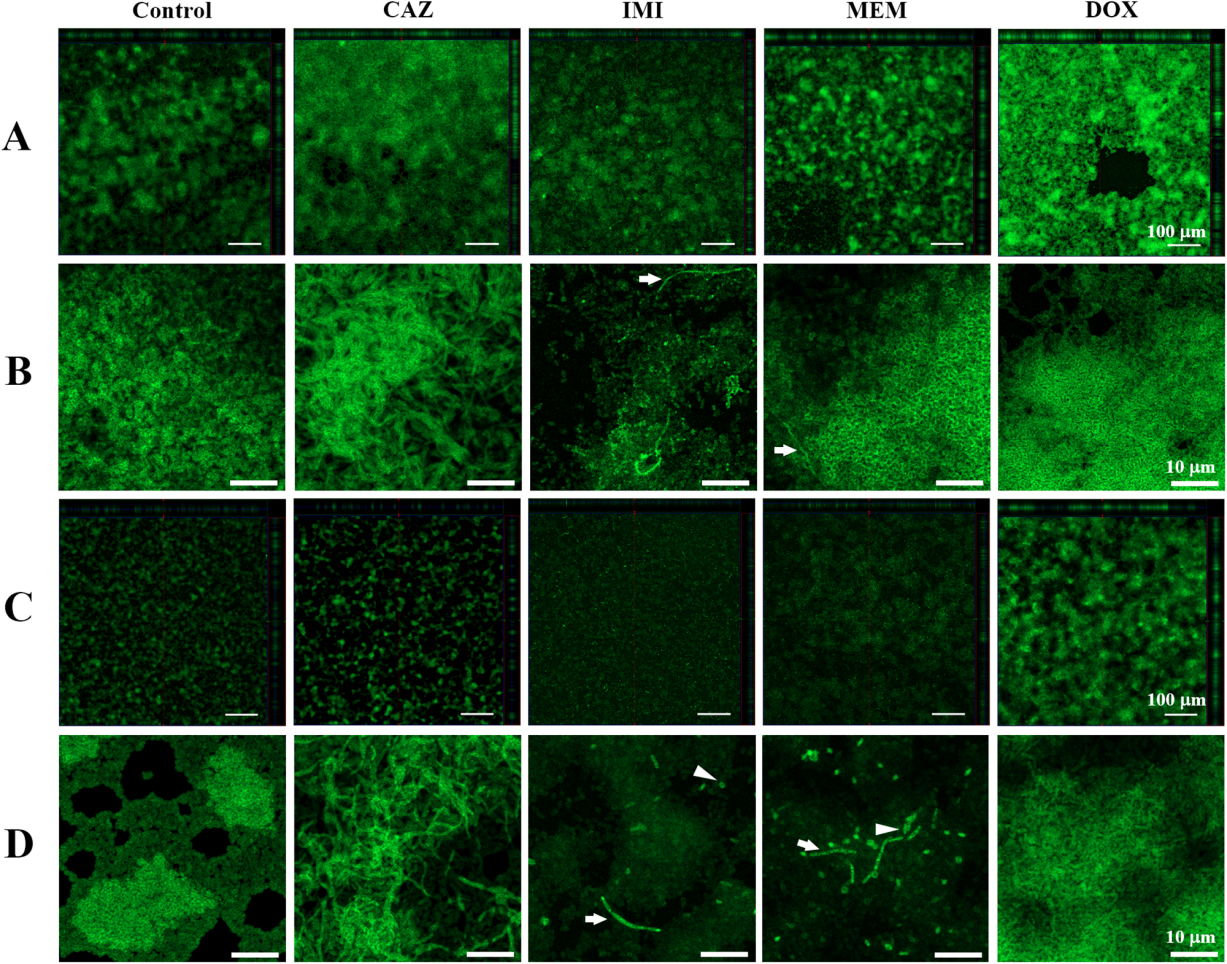
Effect of antibiotics on biofilm structure of *B*. *thailandensis*. CLSM images of 2-day old biofilm of *B*. *thailandensis* E264 exposed to 16×MIC of each antibiotic in MVBM (A, B) and 0.1×MVBM (C, D) for 16 h and stained with fluorescein isothiocyanate-concanavalin A. A representative CLSM image is shown for each sample. At higher magnification (B, D), CAZ-treated biofilm cells displayed a long filamentous change while short filaments (white arrow) and large ovoid cells (white arrow head) were observed for IMI- and MEM-treated biofilm cells similar to those of *B*. *pseudomallei* K96243. The scale bar at 100× magnification (A, C): 100 μm; the scale bar at 630× magnification (B, D): 10 μm.

Fluorescence images of the fluorescein isothiocyanate-concanavalin A-stained 2-day biofilm using CLSM were shown in Figs 3 and 4. Biofilm of *B*. *pseudomallei* and *B*. *thailandensis* in 0.1×MVBM appeared thinner when compared with those in MVBM. These observations were in line with the results of biofilm formation determined by the crystal violet staining method (Fig 2). At low magnification (100×), the structure of *B*. *pseudomallei* and *B*. *thailandensis* biofilm in a MVBM medium of control condition showed honeycomb-like architectures with hollow voids (Figs 3A and 4A) while the biofilm structures in 0.1×MVBM showed a knitted-like structure formed by microcolonies (Figs 3C and 4C). At higher magnification (630×), CAZ-treated biofilms of both strains displayed a long filamentous phenotype (Figs 3B and 4B) while short filaments (white arrow, Figs 3 and 4) and large ovoid morphology (white arrow head, Figs 3 and 4) were observed in IMI- and MEM-treated biofilms of both strains. However, biofilm of both strains treated with DOX showed similar architecture as control in both media.

### Nutrient-limited environment enhances survival of *B*. *pseudomallei* in biofilm

**Fig 5:**
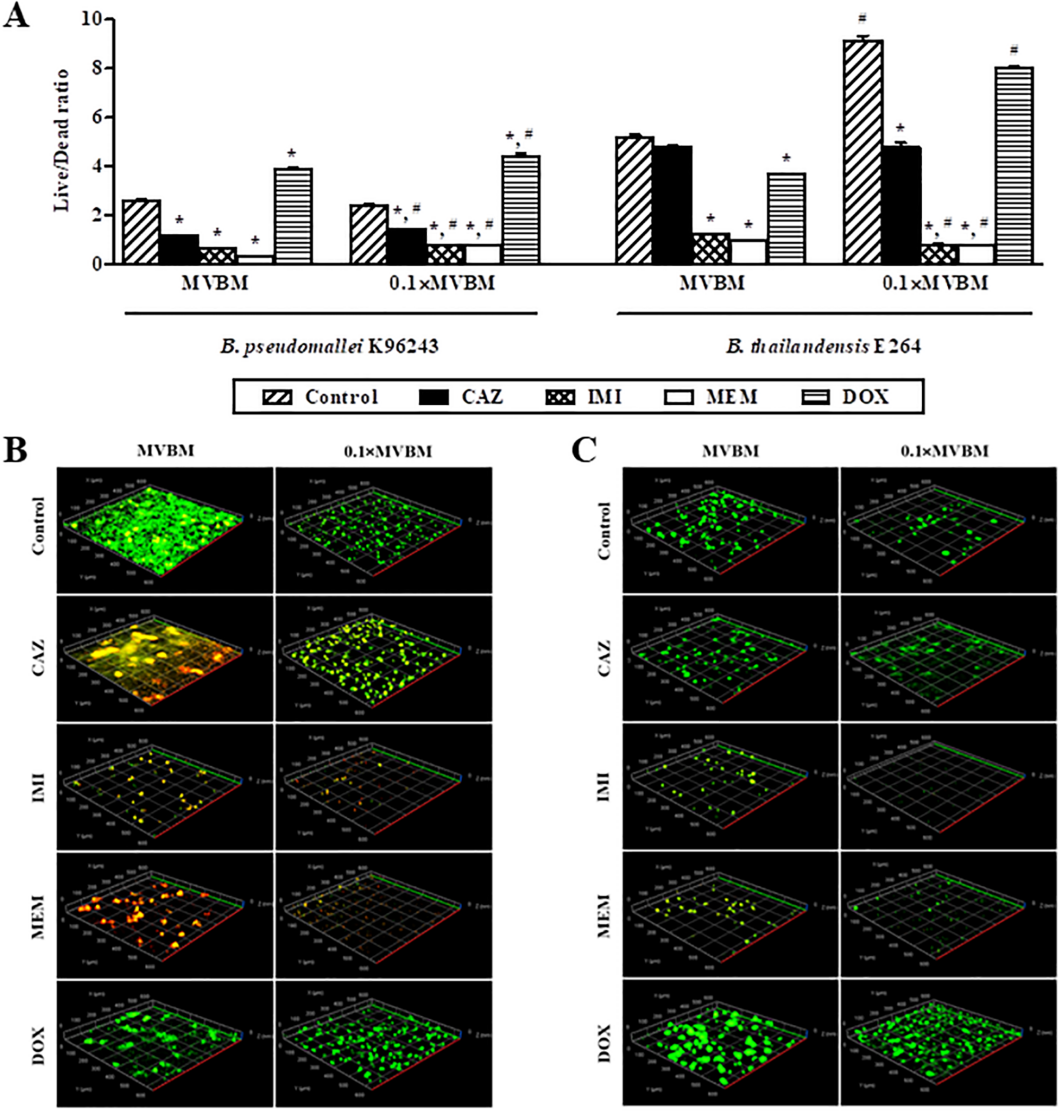
Dead and live cells in 2-day old biofilm of *B*. *pseudomallei* K96243 and *B*. *thailandensis* E264 after treated with 16×MIC of each antibiotic in MVBM and 0.1×MVBM for 16 h. (A) Live/Dead ratios of *B*. *pseudomallei* K96243 and *B*. *thailandensis* E264. Data are the mean value of live/dead ratios from 6 random areas. *p < 0.01 compared to control in the same medium, ^#^*p* < 0.01 compared to the same antibiotic in MVBM. The 3D reconstruction of *B*. *pseudomallei* K96243 (B) and *B*. *thailandensis* E264 (C) biofilm stained with LIVE/DEAD BacLight Bacterial Viability kit; SYTO 9 showing live cells in green and propidium iodide showing dead cells in red (10× objective).

Viability of bacteria in biofilms before and after exposure to the tested antibiotics according to live/dead ratios is shown in Fig 5. For *B*. *pseudomallei* K96243, the number of viable bacteria in 0.1×MVBM after exposure to each antibiotic was significantly higher than in MVBM (*p* < 0.01). Live/Dead ratios of *B*. *pseudomallei* K96243 after exposure to CAZ, IMI, and MEM were significantly lower than control in both MVBM and 0.1×MVBM (*p* < 0.01). MEM exhibited 8-fold and 3-fold higher killing activity compared to untreated controls in MVBM and 0.1×MVBM, respectively (Fig 5A). In contrast, live/dead ratios of *B*. *pseudomallei* K96243 after exposure to DOX were significantly higher than control in both MVBM and 0.1×MVBM (*p* < 0.01). The representative area of *B*. *pseudomallei* K96243 biofilms is shown in Fig 5B. MEM was the most effective antibiotic in killing of *B*. *pseudomallei* in biofilms in both MVBM and 0.1×MVBM followed by IMI and CAZ, respectively. Although the biofilm of *B*. *pseudomallei* in MVBM was thicker than that in 0.1×MVBM, more areas with a high number of dead cells, colored red and yellow were observed compared to biofilm in 0.1×MVBM which has more green areas (Fig 5B). The biofilm of *B*. *pseudomallei* after exposure to DOX showed more live bacteria in green than other antibiotic treatment.

For *B*. *thailandensis* E264, IMI, MEM, and DOX exhibited 4-fold, 5-fold, and 1-fold higher killing activity than control in MVBM, respectively (Fig 5A). Live/Dead ratios of bacteria after exposure to these 3 antibiotics in MVBM were significantly lower than control (*p* < 0.01). For CAZ in MVBM, although lower live/dead ratios of *B*. *thailandensis* were observed, it was not significant difference from control. In 0.1×MVBM, CAZ, IMI, and MEM exhibited 2-fold, 12-fold, and 12-fold significantly higher killing activity than control, respectively (*p* < 0.01). Live/Dead ratio of *B*. *thailandensis* after exposure to DOX in 0.1×MVBM was not significantly different from the control and showed more live bacteria than after other antibiotic treatments (Fig 5A and 5C). Moreover, the number of viable *B*. *thailandensis* after exposure to DOX in 0.1×MVBM was significantly higher compared to DOX in MVBM (*p* < 0.01). From the 3D reconstruction of *B*. *pseudomallei* K96243 (Fig 5B) and *B*. *thailandensis* E264 (Fig 5C) biofilms in both MVBM and 0.1×MVBM, it was observed that IMI and MEM were more potent in destroying the biofilm architecture than CAZ and DOX when compared with control.

### Dynamics of morphological changes in BioFlux microfluidic system

**Fig 6:**
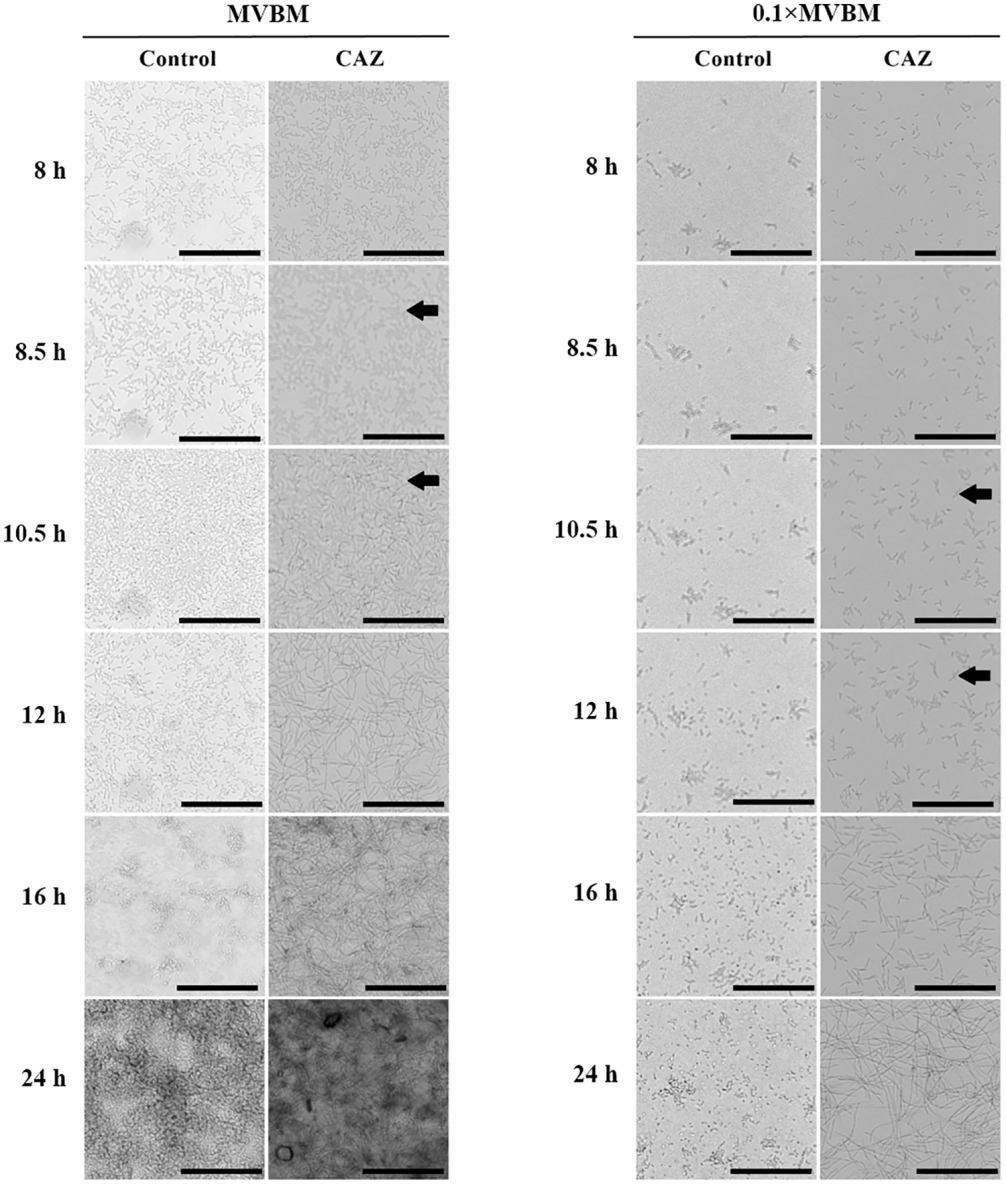
Time-lapse of biofilms under flow conditions. *B*. *thailandensis* E264 biofilm after treated with 16×MIC of CAZ in MVBM and 0.1×MVBM under flow condition using Bioflux microfluidics platform with flow rate 0.5 dyn/cm^2^ at 37 °C for 16 h. Black arrows indicated the starting time when changing of bacterial morphology was observed after treated with CAZ. The scale bar indicates 50 μm. Experiments were performed three times and representative examples are shown. The movies were shown in S1–S4 Figs.

Since CAZ-treated biofilms of both strains showed obvious morphological change into long filaments in static condition, time assessment of morphological changes was evaluated using *B*. *thailandensis* E264 as a model in BioFlux microfluidic system. After bacteria were allowed to form biofilm on glass bottom of the channel for 8 h, CAZ at concentration of 16×MIC was continuously flowed through the channel during 8–24 h. Time-lapse imaging revealed that bacteria started to change their morphology 30 min after exposure to CAZ in MVBM for (black arrow at 8.5 h in Fig 6, and S1 and S2 Figs) and expanded their length gradually. However, in nutrient-limited condition (0.1×MVBM), the bacteria started to change their morphology after 2 h of exposure to CAZ (black arrow at 10.5 h in Fig 6, and S3 and S4 Figs). Under flow condition, biofilms grown in MVBM showed a multilayer structure while dispersed bacteria were found in 0.1×MVBM. The 1-day old biofilm formed in 0.1×MVBM appeared less dense than those in MVBM (S1–S4 Figs).

### Discussion

It is well known that melioidosis is difficult to treat due to the recalcitrant nature with its tendency to recurrence. In addition, the importance of biofilm formation in relapsing melioidosis was reported [7]. From our previous study, biofilm-forming capacity of 50 strains of *B*. *pseudomallei* and 50 strains of *B*. *thailandensis* was evaluated. We found that most of the *B*. *pseudomallei* strains analyzed produced statistically (*P* < 0.01) higher levels of biofilm than *B*. *thailandensis*. However, no correlation could be observed between the biofilm formation and virulence [35]. Therefore, biofilm-forming capacity of *B*. *pseudomallei* is believed to be one of the possible causes of relapsing melioidosis that related to more antibiotic resistant of biofilm bacteria than any virulence factors. However, the dynamics of *B*. *pseudomallei* and *B*. *thailandensis* biofilm formation under conditions with different drugs and nutrient concentrations and its regulation is still mostly unknown. In this study, CLSM was used to characterize and compare *B*. *thailandensis* E264 and *B*. *pseudomallei* K96243 biofilms grown under different drugs and nutrient concentrations. CLSM is useful since it allows visualization of fully hydrated samples revealing 3D structure of biofilms [36] while preserving the EPS structure. From our preliminary study, several subcidal concentrations of antibiotics were tested, i.e. 16×MIC, 64×MIC and 256×MIC. We found that the higher concentrations of antibiotics, the more biofilm architectures were destroyed (data not shown). It is hard to see different effects of 4 antibiotics on biofilm formation and biofilm architectures at higher concentrations of antibiotics (64×MIC and 256×MIC). Therefore, the 16×MIC is the optimal concentration that could demonstrate the different biofilm architectures among 4 antibiotics. The results demonstrated that using MVBM medium or 10-fold diluted MVBM provided completely different results, particularly regarding the structure of *B*. *thailandensis* E264 and *B*. *pseudomallei* K96243 biofilms. As described above, the structure of *B*. *pseudomallei* biofilm in control condition changed from honeycomb-like architecture in a MVBM medium to a knitted-like structure formed by microcolonies in a nutrient-limited medium 0.1×MVBM. Such different structures have been previously described for other species, including *L*. *monocytogenes*, and *Staphylococcus aureus* [22,37,38]. Moreover, the nutrient content of the medium and type of antibiotics significantly influenced the quantity of produced biofilm. MEM and IMI are able to decrease *B*. *pseudomallei* biofilm formation better than CAZ and DOX (Fig 2) and caused significantly more damage to the architecture of *B*. *pseudomallei* biofilms (Figs 3 and 5). In addition, killing activities of MEM and IMI were higher than of CAZ and DOX on *B*. *pseudomallei* both in planktonic cells and in 2-day old biofilm (Tables 1 and 2, Fig 5). Similar results were observed for *B*. *thailandensis*. These results could explain why the outcome of treatment failure was significantly more common in patients treated with CAZ than IMI (41.3% compared with 20.3%) in previous clinical study [39].

In this study, CAZ-treated biofilms of *B*. *pseudomallei* and *B*. *thailandensis* in both MVBM and 0.1×MVBM displayed a change from rod-like morphology to a filamentous structure (Figs 3 and 4, and S1–S4 Figs). These results could be due to the ability of CAZ to induce filamentation by binding to penicillin-binding protein 3, which is required for the synthesis of septa during cell division [40–42]. Filamentation occurs when cell growth continues in the absence of cell division, and results in the formation of elongated organisms. It has been suggested that filamentation is an adaptive response used by bacteria to increase survival under a range of stress conditions like limited nutrients or during antibiotic exposure [43]. These may be the reason why the long filamentous structure of bacteria demonstrated less susceptibility to CAZ than IMI and MEM.

IMI and MEM were reported to inhibit cell wall synthesis by acting on penicillin-binding protein 2 [42]. In the present study, short filamentous and large ovoid morphologies were observed for bacteria treated with IMI and MEM. No long filamentous structure was found. Our results are in accordance with several studies for other species, *i*.*e*. *Klebsiella pneumonia* and *Acinetobacter baumannii*, which reported that IMI-treated bacteria changed their morphology into large ovoid structures [44–46]. The effect of IMI and MEM on bacterial morphology which was different from CAZ-treated bacteria may be correlated with their affinities for different penicillin-binding protein.

It has been previously noted that subinhibitory concentrations of some antibiotics can stimulate bacterial biofilm formation [47,48]. Such phenomenon was clearly observed for DOX-treated bacteria in this study. After exposure to DOX, increased biofilm formation was seen compared to untreated controls except for culture condition in MVBM (Fig 2). DOX inhibits growth of bacteria by binding to the 30S ribosomal subunit thus inhibiting protein synthesis [49]. This working mechanism of DOX is different from CAZ, IMI and MEM. No filamentation was found after bacteria were exposed to DOX. These results are consistent with a previous study by Chen *et al*. [40]. In addition, no differences in biofilm structure after treated with DOX compared to control was observed. Moreover, the biofilm treated with DOX showed higher live/dead ratio than the other tested antibiotics (Fig 5). This might explain, at least in part, the high rate of treatment failure after treated with DOX in patients [8].

In the present study, there are two different stresses being evaluated—antibiotic treatment and low nutrient stress. It is well accepted that both of these stresses are capable of activating bacterial cell responses, such as the stringent response and bacterial persistence or tolerance. From our results, it is possible that there is a synergistic effect between the two stresses, however further studies are needed to proof whether these effects are synergistic.

In conclusion, this study provided more insight into the biofilm architecture of *B*. *pseudomallei* and *B*. *thailandensis* under different antibiotics and nutrient concentrations. The findings of this study indicate that nutrient-limited environment enhanced survival of *B*. *pseudomallei* in biofilm after exposure to the tested antibiotics. The shedding planktonic *B*. *pseudomallei* and *B*. *thailandensis* were also found to increase CAZ tolerance in nutrient-limited environment. However, killing activities of MEM and IMI are stronger than CAZ and DOX on *B*. *pseudomallei* and *B*. *thailandensis* both in planktonic cells and in 2-day old biofilm. In addition, MEM and IMI are able to decrease *B*. *pseudomallei* biofilm formation better than CAZ and DOX. Since nutrient-limited environment normally occurs in biofilm and biofilm formation is believed to be one of the possible causes of relapse, MEM and IMI may be better therapeutic options than CAZ and DOX for melioidosis treatment.

## Supporting information

S1 Fig Morphology of *B*. *thailandensis* E264 in MVBM at 8–24 h. (AVI)

S2 Fig Morphology of *B*. *thailandensis* E264 after treated with 16×MIC CAZ in MVBM at 8–24 h. (AVI)

S3 Fig Morphology of *B*. *thailandensis* E264 in 0.1×MVBM at 8–24 h. (AVI)

S4 Fig Morphology of *B*. *thailandensis* E264 after treated with 16×MIC CAZ in 0.1×MVBM at 8–24 h. (AVI)
